# Highly Versatile Upconverting Oxyfluoride-Based Nanophosphor Films

**DOI:** 10.1021/acsami.1c07012

**Published:** 2021-06-18

**Authors:** Thi Tuyen Ngo, Elena Cabello-Olmo, Encarnación Arroyo, Ana I. Becerro, Manuel Ocaña, Gabriel Lozano, Hernán Míguez

**Affiliations:** Instituto de Ciencia de Materiales de Sevilla, Consejo Superior de Investigaciones Científicas-Universidad de Sevilla, Américo Vespucio 49, 41092, Sevilla, Spain

**Keywords:** light-emission, upconversion, nanoparticles, rare-earth nanomaterials, multifunctional coatings, flexible materials

## Abstract

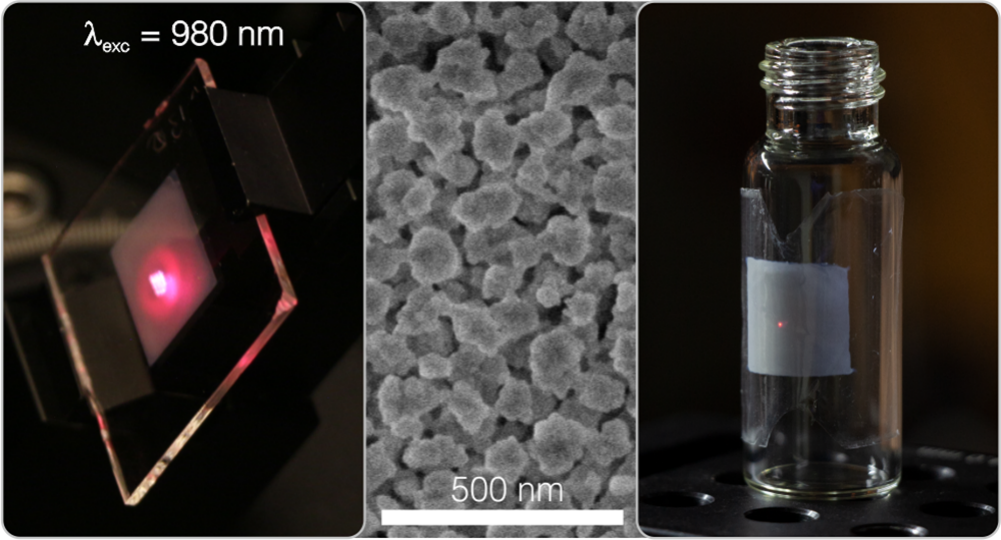

Fluoride-based compounds doped with rare-earth cations are the preferred choice of materials to achieve efficient upconversion, of interest for a plethora of applications ranging from bioimaging to energy harvesting. Herein, we demonstrate a simple route to fabricate bright upconverting films that are transparent, self-standing, flexible, and emit different colors. Starting from the solvothermal synthesis of uniform and colloidally stable yttrium fluoride nanoparticles doped with Yb^3+^ and Er^3+^, Ho^3+^, or Tm^3+^, we find the experimental conditions to process the nanophosphors as optical quality films of controlled thickness between few hundreds of nanometers and several micrometers. A thorough analysis of both structural and photophysical properties of films annealed at different temperatures reveals a tradeoff between the oxidation of the matrix, which transitions through an oxyfluoride crystal phase, and the efficiency of the upconversion photoluminescence process. It represents a significant step forward in the understanding of the fundamental properties of upconverting materials and can be leveraged for the optimization of upconversion systems in general. We prove bright multicolor upconversion photoluminescence in oxyfluoride-based phosphor transparent films upon excitation with a 980 nm laser for both rigid and flexible versions of the layers, being possible to use the latter to coat surfaces of arbitrary shape. Our results pave the way toward the development of upconverting coatings that can be conveniently integrated in applications that demand a large degree of versatility.

## Introduction

Upconversion (UC) photoluminescence is a nonlinear optical phenomenon by which a material emits light at higher frequency than the one used for excitation, typically in the near-infrared (NIR). This way, nonvisible light is converted into the visible region of the electromagnetic spectrum.^1−4^ This phenomenon is of great interest for a wide variety of research fields, including bioimaging,^5−7^ optogenetics,^8^ super-resolution microscopy,^4,9^ light guiding,^10^ light harvesting,^11−15^ color displays,^16^ or sensing.^17,18^ UC luminescent materials are generally phosphors, i.e., inorganic hosts doped with rare-earth (RE) cations, e.g., Er^3+^, Ho^3+^, and Tm^3+^, with unique ladderlike energy levels. In the canonical example, green and red light is obtained exciting in the NIR using Er^3+^ as an active cation and Yb^3+^ as a sensitizer to improve conversion efficiency,^18−20^ and thus the brightness of the process. Nevertheless, quenching mechanisms associated with impurities, defects, or energy migration in highly doped samples pose reasonable doubts about the prospects of these materials.^21^

Indeed, the major challenge to develop applications based on UC nanotechnology is related to efficiency and brightness.^22,23^ Several strategies to overcome above-mentioned issues have been designed, which include surface passivation through core–shell architectures, engineering the distribution of dopants or choosing the right host for the nanophosphor.^2,24,25^ Most efficient UC nanophosphors consist of fluoride-based hosts, which feature very low phonon energies that decrease the probability of nonradiative paths through multiphonon relaxation. Specifically, sodium yttrium fluoride (NaYF_4_) stands out as the most efficient host developed to date, despite featuring low thermal stability,^3,19,26^ which limits its applicability. Conversely, oxide hosts typically feature better thermal stability, although these matrices come with higher phonon energies, being therefore inclined to suffer from lower UC efficiencies.^27,28^ Therefore, it remains intriguing to develop host nanomaterials that combine the robustness of oxides with the high conversion efficiencies associated with fluorides. In this context, oxyfluorides represent a promising family of hosts, which has generated interest in recent years.^29−31^

Along with the quest for stable, bright and efficient materials, scientists are lately concerned about their processing, required to achieve versatile UC coatings that can be readily integrated in devices that benefit from them.^11,16,32^ Indeed, features beyond efficiency such as transparency, pliability or tailor-made chromaticity are sought after.^33−39^ Thus, flexible and transparent polymer waveguides based on UC nanophosphors have been demonstrated by dispersing UCNPs in a polymer.^10,40^ However, it is still challenging to increase nanophosphor filling fraction without compromising the stability of the composite. Besides, multicolor emission is generally achieved by doping a single nanomaterial with different cations.^41,42^ Yet, activator codoping typically leads to unwanted cross-relaxation that results in UC quenching, along with a strong dependence of the color on the excitation power.^43^

In this work, we demonstrate multicolored, transparent, and adaptable UC oxyfluoride-based films. We report a simple method to develop nanophosphor pastes to fabricate optical quality films of controlled thickness from YF_3_:Yb^3+^,X^3+^ (X = Er, Ho, or Tm) nanoparticles synthesized at low temperature, following a solvothermal route. Rigid rare-earth (RE)-doped oxyfluoride (REOF) films were obtained by transforming YF_3_ to oxyfluoride by thermal annealing in air. Self-standing flexible REOF films were also proved by infiltrating the porosity of the rigid layers with poly(methyl methacrylate) (PMMA). Structural and photophysical properties of both rigid and flexible versions of the coatings were thoroughly analyzed to find the processing conditions that yield the brightest UC. Indeed, red, orange, and blue UC photoluminescence was observed by exciting REOF films with a 980 nm laser. Finally, the versatility of the coatings allowed us to demonstrate UC white light by preparing a stack of orange- and blue-emitting layers.

## Results and Discussion

### Fluoride-Based Films

**Figure 1:**
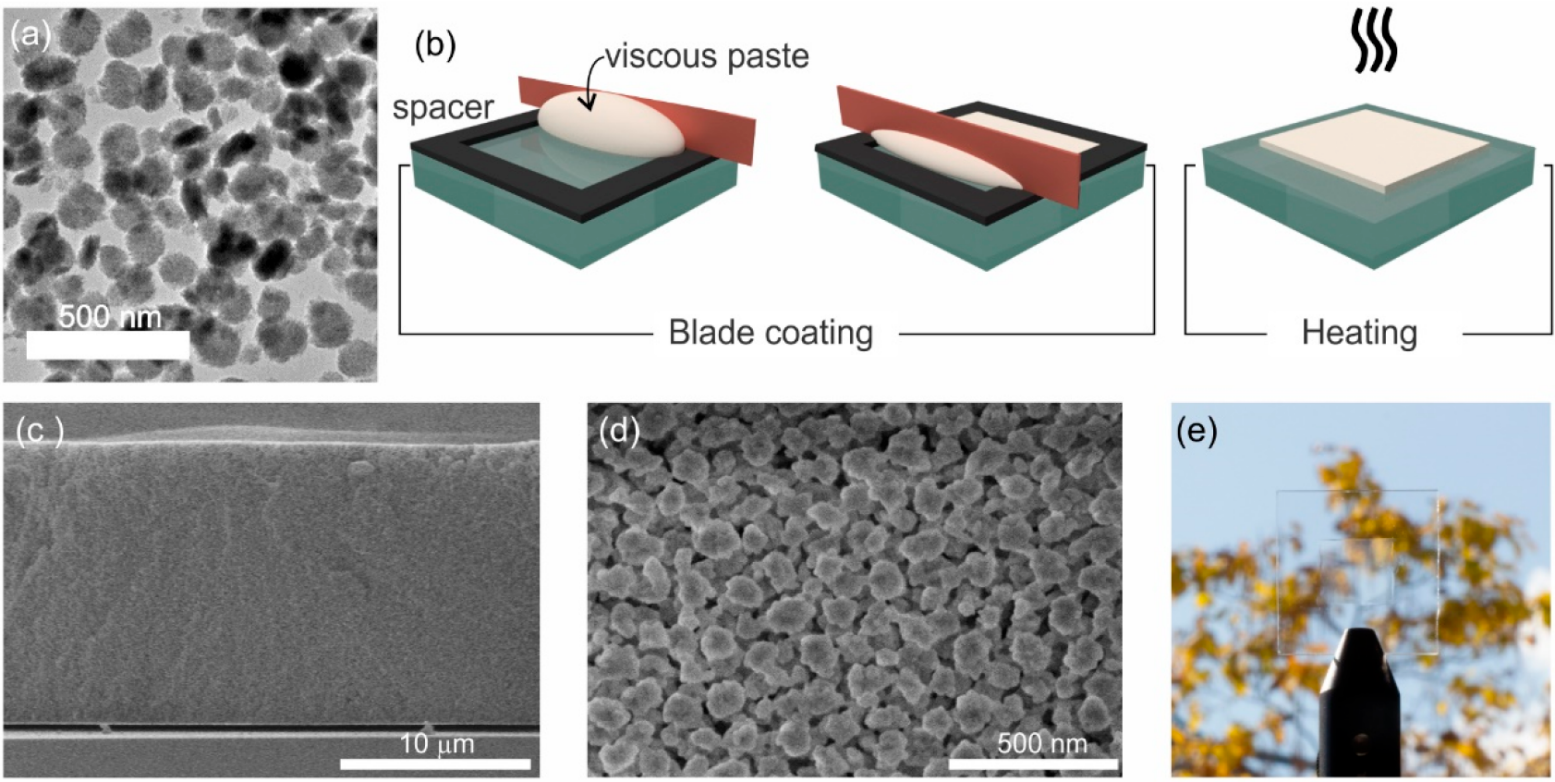
(a) Transmission electron microscopy (TEM) micrograph of the starting YF_3_:Yb^3+^Er^3+^ nanoparticles. (b) Schematic processing of nanophosphor film preparation by doctor blading method from a nanophosphor paste. (c, d) Scanning electron microscopy (SEM) micrographs of a cross section (panel (c)) and a top view (panel (d)) of a nanophosphor film annealed at 450 °C for 6 h. (e) Picture of the same film under sunlight.

Figure 1a displays a transmission electron microscopy (TEM) image of as-synthesized YF_3_:Yb^3+^,Er^3+^ nanoparticles, which are uniform and present an ellipsoid-like morphology, with a mean long dimension of 134 nm ± 19 nm (see Figure S1 in the Supporting Information for more details).^44^ In order to produce phosphor films, we developed nanocrystal-based pastes by mixing the nanophosphors with an organic binder.^45,46^ Full details are provided in the Methods and Materials section. Transparent flat films were deposited over quartz substrates by blade coating and heated at different temperatures, following the steps schemed in Figure 1b. This method allows the preparation of mechanically stable and uniform films with thicknesses ranging from ∼1 μm to ∼15 μm.^45^ Notice, it is also possible to deposit nanophosphor films with thickness below ∼1 μm from the spin coating of nanoparticle suspensions in volatile solvents,^47−49^ as detailed in the Methods and Materials section and shown in Figure S2 in the Supporting Information. Thermal annealing is used not only to remove any organic impurities from the nanoparticle synthesis or the paste, and provide mechanical stability to the film, but also to study the interplay between structural and photophysical properties of the light-emitting layers. As an example, Figures 1c and 1d show scanning electron microscopy (SEM) images of the cross section and top view of 14.7-μm-thick (Yb^3+^, Er^3+^)-doped nanophosphor film annealed at 450 °C for 6 h. Our method leaves a connected nanocrystal network, as it can be clearly observed in Figure 1d, with a filling fraction of ∼50% after the organic part is removed. Pictures reveal that nanophosphors show similar size and shape after annealing, with transparency being fully preserved, as shown in Figure 1e.

### Upconversion Photoluminescence and Transparency

**Figure 2:**
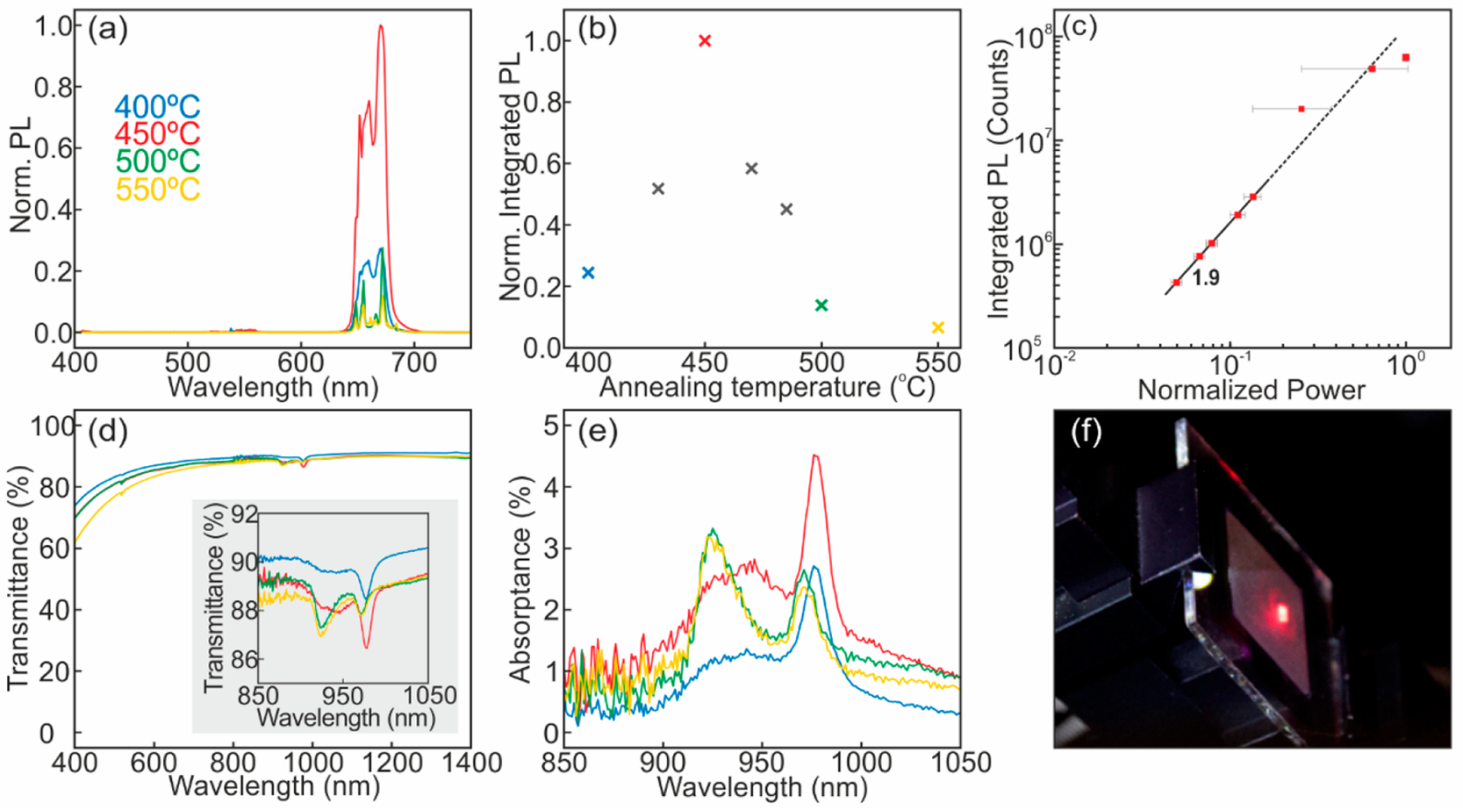
(a) Upconversion photoluminescence (UCPL) spectra and (b) spectrally integrated UCPL intensity of nanophosphor films annealed for 6 h at different temperatures. Integral is performed between 400 nm and 750 nm. (c) Integrated PL intensity of the red emission (between 640 nm and 700 nm), as a function of the excitation power for the nanophosphor film annealed at 450 °C. PL measurements were performed using a 980 nm continuous wave laser as excitation source operating at full power, and a computer-controlled neutral density filter wheel to attenuate the output of the laser. Uncertainty in the excitation power values originates from the uneven nature of the attenuation factor provided by the filter. (d) Total transmittance and (e) absorptance spectra measured from films annealed at different temperatures, as labeled in panel (a). (f) Picture of the UCPL of a (Yb^3+^, Er^3+^)-doped nanophosphor film.

UC photoluminescence (UCPL) spectra of (Yb^3+^, Er^3+^)-doped nanophosphor films with a thickness of ∼15 μm annealed at temperatures ranging from 400 °C to 550 °C for 6 h are shown in Figure 2a. 980 nm laser light is absorbed by the Yb^3+^ ions and energy is transferred between ^2^F_5/2_ and ^4^I_11/2_ levels of Yb^3+^ and Er^3+^, respectively. Eventually Er^3+^ cations relax to the ground-state emitting light.^2,3^ Specifically, the most intense emission is observed in the red part of the electromagnetic spectrum, between 640 nm and 700 nm, and originates from the ^4^F_9/2_ – ^4^I_15/2_ transition of Er^3+^. Much weaker green and blue emission bands, as shown in Figure S3 in the Supporting Information, associated with ^4^S_3/2_, ^2^H_11/2_, and ^2^H_9/2_ transitions to the ground state ^4^I_15/2_ are also identified.^50−52^ Interestingly, we observe a ∼5-fold increase of UCPL intensity with the annealing temperature from 400 °C to 450 °C, whereas using higher temperatures turns out to be detrimental for the UCPL intensity, as illustrated by the ∼10-fold decrease from 450 °C to 550 °C displayed in Figure 2b. Generally, thermal annealing improves PL quantum yield by removing lattice defects and eliminating quenching pathways caused by organics from the synthesis. Nevertheless, temperature might also induce a transformation of the host material from fluoride to oxide transiting through oxyfluoride.^53−55^ From this perspective, the transition toward yttrium oxide results in a host lattice featuring phonons with significantly higher energy, which is highly detrimental for UCPL. The nonlinear nature of the emission is illustrated in Figure 2c for the film annealed at 450 °C. In particular, we show the integrated UCPL for the ^4^F_9/2_–^4^I_15/2_ transition of Er^3+^, e.g., from 640 nm to 700 nm, as a function of the excitation power. Please see Figure S3 in the Supporting Information for the analysis of the power dependence of green band. Films annealed at other temperatures feature the same behavior. Measurements indicate that UCPL increases with the excitation power in two distinct ranges, depending on the depopulation mechanism of the ^4^I_11/2_ intermediate energy level of Er^3+^. Hence, UCPL dynamics is dictated by the competition between two different phenomena: (i) energy transfer upconversion (ETU) rate from ^4^I_11/2_ intermediate-energy level to high-energy levels of Er^3+^, and (ii) relaxation from ^4^I_11/2_ to the ground state.^56^ In the low power regime, intermediate state depopulation dominates over ETU and UCPL exhibits a quadratic dependence associated with the sequential absorption of two photons. In this range, our measurements are fitted with a slope of ∼1.9, as shown in Figure 2c. Above a certain energy threshold, the ^4^I_11/2_ level saturates, because of the fast energy transfer rate from Yb^3+^ sensitizer and UCPL is achieved by the absorption of only one photon, following a linear power dependence.^57−59^ Analysis shows a change in the power dependence of the UCPL for excitation power values above 20%–60% of the maximum power employed, with a clear reduction of the slope. However, we do not count enough experimental data in this high-energy range to confirm the linear power dependence expected.

In order to evaluate the transparency of the layers, we performed optical transmittance measurements. Indeed, total transmittance spectra of (Yb^3+^, Er^3+^)-doped nanophosphor films, with a thickness of ∼15 μm, are displayed in Figure 2d. (Please check Figure S4 in the Supporting Information for reflectance measurements.) Despite their large thickness, all layers are fairly transparent in the visible range, with values of ∼90% from far-red on, as shown in Figure 2d. Yet, transmittance reduces up to 70% at short wavelengths, especially for films annealed at high temperature, suggesting a larger fraction of scattered light, due to some nanoparticle clustering.^47^ It is noteworthy that transparency values attained surpass that of any previous report,^1,34,60,61^ and similar figures have been only observed in extremely thin layers (∼100 nm),^62^ or in polymer films with low filling fraction of nanophosphors.^10,33^ Importantly, transmittance of our UC films feature a dip at ∼950 nm, as shown in the inset of Figure 2d. Indeed, absorptance measurements displayed in Figure 2e confirm absorption bands associated with the ^2^F_7/2_–^2^F_5/2_ transition of Yb^3+^ ions at ∼950 nm.^50,63−67^ Specifically, a clear peak is observed at ∼980 nm, where we excite the films, revealing the underlying UCPL mechanism. The appearance of several absorption peaks demonstrates the existence of sub-energy levels associated with the ^2^F_7/2_ and ^2^F_5/2_ transitions, as it has been discussed elsewhere.^68,69^ Different annealing conditions yield slight variations in the position of the absorption bands, their width, and relative intensity, which brings to light the influence of the host matrix and the local environment of Yb^3+^ ions in the optical response of the material. We attain values of the fraction of the incident light at 980 nm absorbed by (Yb^3+^, Er^3+^)-doped nanophosphor films comprised between 1.6% and 3.4%, depending on the annealing conditions. Therefore, differences observed in UCPL—shown in Figures 2a and 2b—must originate mainly from variations in the efficiency of the emission process itself, as it will be discussed next. These results demonstrate a transparent upconverting layer based on nanosized phosphors, enabling strong red UCPL when excited in the NIR, as depicted in Figure 2f. The development of transparent luminescent films with thickness on the order of the wavelength opens the door to their combination with photonic architectures specifically designed to modify UC emission.

### Crystalline Phase and Time-Dependent Photoluminescence

**Figure 3:**
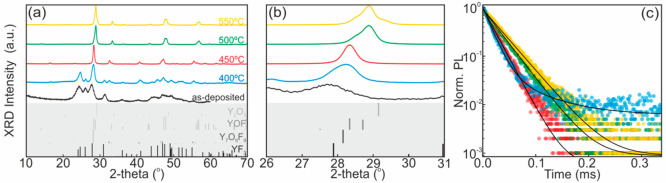
(a) X-ray diffraction (XRD) patterns of (Yb^3+^, Er^3+^)-doped nanophosphor films annealed at different temperatures for 6 h. (b) Zoom of the angular range showing the most intense peak displayed in panel (a). Powder Diffraction File (PDF) reference codes of the patterns included are 00-032-1431 for YF_3_, 01-070-0867 for Y_7_O_6_F_9_, 00-025-1012 for YOF, and 00-041-1105 for Y_2_O_3_. (c) Time-dependent UCPL of films annealed at different temperatures (blue circles for 400 °C, red for 450 °C, green for 500 °C, and yellow for 550 °C), along with their fittings (shown in black). A 980 nm pulsed laser was used as an excitation source. Emission is collected at 670 nm.

In order to analyze the structural properties of (Yb^3+^, Er^3+^)-doped nanophosphor films, we performed X-ray diffraction (XRD) measurements. Full details are provided in the Methods and Materials section. Figure 3a displays XRD patterns for ∼15 μm-thick films annealed at 400, 450, 500, and 550 °C. For comparison, we show the diffractogram of a thin film (∼1 μm) of (Yb^3+^, Er^3+^)-doped nanophosphors (labeled as “as-deposited”), which feature the orthorhombic YF_3_ phase (Powder Diffraction File (PDF) No. 00-032-1431).^44^ Paste-blading requires thermal processing for film mechanical stabilization, which makes it unfeasible to perform any structural or optical characterization on as-prepared layers. For this reason, we use a film deposited from a suspension of the same YF_3_:Yb^3+^,Er^3+^ nanoparticles, which does not require any thermal processing to acquire mechanical stability, to compare XRD patterns of upconverting films annealed at different temperatures with that of an as-prepared sample, i.e., devoid of any thermal processing. Annealing induces a phase transformation of the nanophosphor matrix, because of the inherent instability of the fluoride host. Indeed, O atoms replace F ones in the lattice, increasing the oxygen content with temperature and time, until complete oxidation of YF_3_ into Y_2_O_3_ is eventually fulfilled.^31,54^ Specifically, films annealed at 400 °C show a two-phase mixture of orthorhombic YF_3_ and orthorhombic Y_7_O_6_F_9_ (PDF No. 01-070-0867). Increasing the annealing temperature to 450 °C turns the film into a pure orthorhombic Y_7_O_6_F_9_ phase. In turn, samples annealed at 500 °C possess a pure rhombohedral YOF phase (PDF No. 00-025-1012), whereas at 550 °C, a two-phase mixture of rhombohedral YOF and cubic Y_2_O_3_ phase (see PDF No. 00-041-1105) is found. Notice that experimental patterns appear to be displaced to higher angles, with respect to the theoretical ones for all cases, which can be attributed to the effect of doping cations on the size of the unit cell. Substitution of Y^3+^ ions for smaller Er^3+^ and Yb^3+^ in the lattice results in unit-cell contraction, causing a shift of the reflections to higher diffraction angles.

Kinetics of the transition allows attaining different phase mixtures at a given temperature, depending on the duration of the thermal processing (see Figure S5 in the Supporting Information). In addition to the phase transformation, at 450 °C, a significant increase in the XRD intensity of the peaks is clearly observed, along with a reduction in their full width at half-maximum, which indicates crystallinity was improved as a result of the annealing. Besides healing crystal lattice defects, thermal processing enables the removal of organics that act as quenchers of the emission. All together, these effects result in the rise of the UCPL for samples annealed between 400 and 450 °C shown in Figures 2a and 2b. However, further increase in the annealing temperature leads to nanophosphor matrices with higher oxygen content and higher phonon energy, which cause a rapid reduction in the UCPL,^27,28,70,71^ as displayed in Figures 2a and 2b. Our results bring out an inherent tradeoff in the quest of efficient oxyfluoride materials: annealing temperature must be high enough to remove organic quenchers and heal lattice defects but at the same time temperature must be kept as low as possible to limit the oxidation of the fluoride host.

Local environment of Er^3+^ emitters determines the dynamics of the UCPL. We analyze time-dependent UCPL of films annealed at temperatures ranging from 400 °C to 550 °C to stablish a clear relationship between lifetime and crystal structure. We monitor the most intense Er^3+^ transition, i.e., ^4^F_9/2_–^4^I_15/2_. Results are shown in Figure 3c and Table 1. A two-exponential model
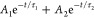
1is employed to describe the PL dynamics of nanophosphors to account for the two different decay rates (τ_1_^–1^ and τ_2_^–1^) expected for Er^3+^ cations embedded in different crystal lattices. A_1_ and A_2_ are fitting constants associated with the relative weight *w*_*i*_ of each contribution to the sum. A similar model has been proven useful to account for the different rates expected for cations located in the bulk or close to the surface of smaller (<50 nm) nanophosphors.^47^ Our analysis reveals two distinct components for films annealed below 450 °C, in which a two-phase mixture of Y_7_O_6_F_9_ and YF_3_ was identified, with a high decay rate component τ_1_^–1^ ≈ (20 μs)^−1^ associated with the Y_7_O_6_F_9_ phase, which is predominant (relative weight above 90%), and a low decay component τ_2_^–1^ ≈ (67 μs)^−1^ associated with Er^3+^ cations that sit in the YF_3_ lattice. We associate the low decay component with the fluoride phase, because lifetimes connected to fluoride lattices are generally longer than those of oxyfluorides.^19,72,73^ Small variations around these average values are observed (see Table 1). These are expected considering that photoluminescence dynamics is extremely sensitive to the local environment of the emitters, which can be slightly different, depending on the processing conditions. Interestingly, the relative contribution of the low decay rate component decrease as the annealing temperature increases, which is in agreement with the gradual oxidation of the YF_3_ phase, as previously discussed. In turn, a single exponential model describes the UCPL dynamics of films annealed at 450 °C or higher due to the complete annihilation of the fluoride phase. Such single exponential character is also found for films annealed at 470 and 485 °C, for which we assign a mixture of oxyfluoride crystalline phases. This could be due to the fact that there are no significant differences between the decay rate values associated with the different oxyfluoride phases. It is noteworthy that the value of the decay rate τ_1_^–1^ remains barely unaltered up to 485 °C, as a consequence of the presence of the Y_7_O_6_F_9_ crystal phase. This decay rate changes, to ∼(30 μs)^−1^ and ∼(35 μs)^−1^, when thermal annealing induces phase transformations at 500 and 550 °C toward rhombohedral YOF, in agreement with previous reports.^30,31^ Our results stablish a precise correlation between lifetime and crystal phase transition that can be exploited in the optimization of oxyfluoride-based systems.

**Table 1:** Fitting Parameters of the Time-Dependent UCPL Measured from Nanophosphor Films Annealed at Different Temperatures along with the Corresponding Crystal Phase of the Host in Each Case, As Extracted from the XRD Analysis

annealing temp, *T* (°C)	τ_1_ (μs)	*w*_1_ (%)	τ_2_ (μs)	*w*_2_ (%)	host crystal phase
400	15.6	93.7	68.3	6.3	YF_3_ + Y_7_O_6_F_9_
430	21.2	95	64.9	5	YF_3_ + Y_7_O_6_F_9_
450	21.2	100	–	–	Y_7_O_6_F_9_
470	19.5	100	–	–	Y_7_O_6_F_9_ + YOF
485	20.2	100	–	–	Y_7_O_6_F_9_ + YOF
500	30.5	100	–	–	YOF
550	34.4	100	–	–	YOF + Y_2_O_3_

### Flexible Upconverting Films

**Figure 4:**
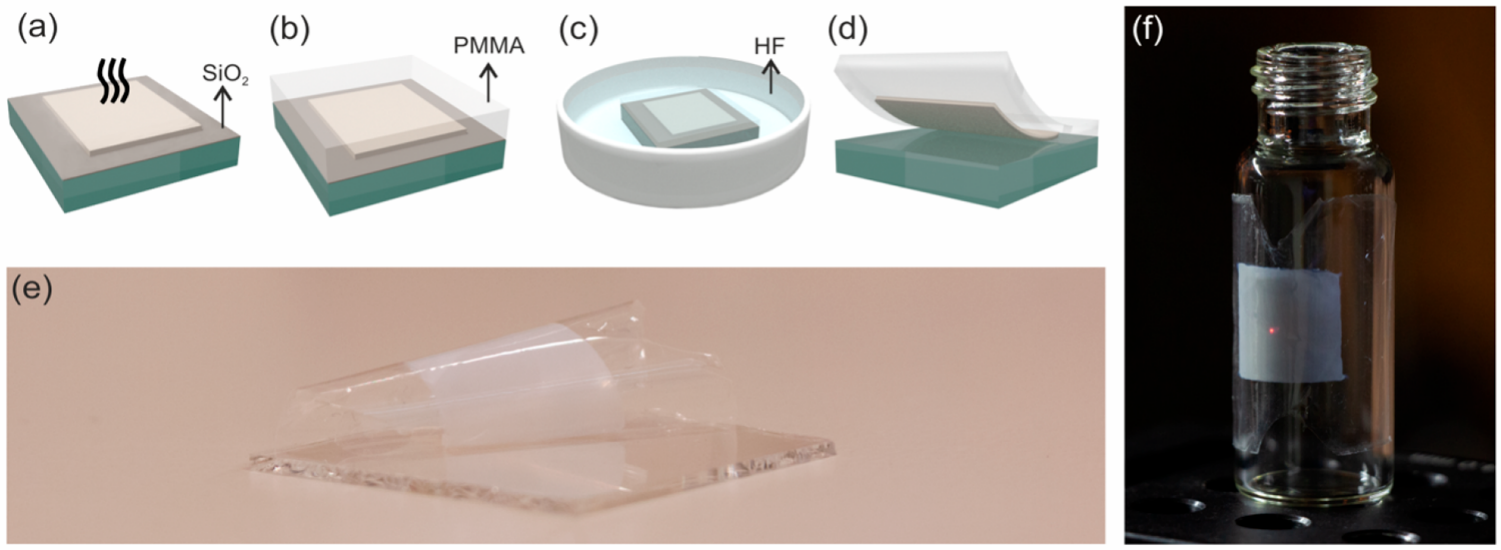
(a-d) Schematic processing of the preparation of UC flexible films: nanophosphor film deposition and annealing (a), polymer infiltration (b), acid immersion (c) and lift-off (d). (e) Digital picture of a flexible (Yb^3+^, Er^3+^)-coating detached from its rigid substrate. (f) Digital picture of the same coating placed over the curved surface of an empty glass vial under the excitation with a 980 nm continuous wave laser.

In order to demonstrate the versatility of our method, we prepare self-standing flexible UCPL films. We take advantage of the porosity of the nanoparticle-based oxyfluoride film to infiltrate the pore network with a polymer that bestows the coating with new mechanical properties. In brief, we deposit an ∼5-μm-thick nanophosphor film on a thin sacrificial layer made of SiO_2_, following the procedure described in the Methods and Materials section. After annealing, the porous nanophosphor film is infiltrated with PMMA and the resulting composites present enough mechanical stability to be lifted off. Specifically, the SiO_2_ layer was removed by immersing the sample in hydrofluoric acid solution, detaching the nanophosphor/PMMA composite from the rigid substrate. Each step of the process is shown schematically in Figure 4a–d. As a result, a flexible nanophosphor film (see picture in Figure 4e) is attained. The new functionality allows the transferring of the films to coat surfaces of arbitrary shape. This is illustrated in Figure 4f, in which we show the UCPL of a PMMA-infiltrated (Yb^3+^, Er^3+^)-doped nanophosphor film. The flexible coating adapts to the curved surface of a glass vial and shines red light when excited with a 980 nm laser, similar to its rigid counterpart. In fact, the infiltration of PMMA does not alter neither the spectral content nor the dynamics of the UC emission (see Figure S7 of the Supporting Information for more details).

### Tunable Upconversion Chromaticity

**Figure 5:**
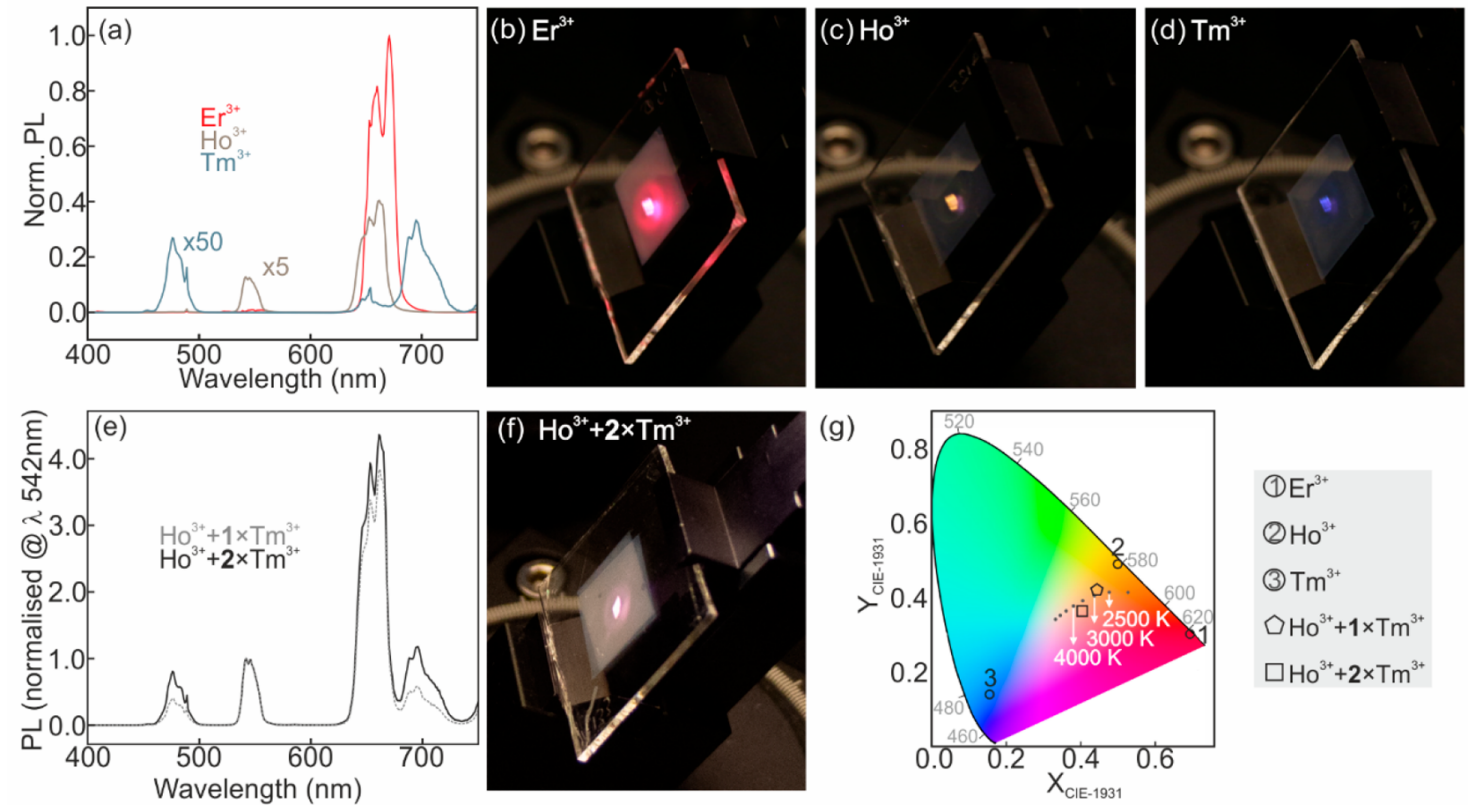
(a) Upconversion photoluminescence (UCPL) spectra of (Yb^3+^, Er^3+^)-, (Yb^3+^, Ho^3+^)- and (Yb^3+^, Tm^3+^)-composite films plotted with red, gray, and blue curves, respectively. (b–d) Digital camera pictures for the UCPL of (Yb^3+^, Er^3+^) (panel (b)), (Yb^3+^, Ho^3+^) (panel (c)), and (Yb^3+^, Tm^3+^)-doped nanophosphor films infiltrated with PMMA (panel (d)) under excitation with 980 nm laser light. (e) UCPL spectra of stack comprising a (Yb^3+^, Ho^3+^)-doped composite layer plus one (light gray curve) or two flexible (Yb^3+^, Tm^3+^)-coatings (dark gray curve). (f) Digital camera picture of the UCPL of the latter under excitation with 980 nm laser light. (g) Color coordinates in a CIE 1931 chromaticity diagram of UCPL spectra shown in panels (a) as circled numbers and (e) as pentagon and square symbols. Correlated color temperatures associated with the Planckian locus between 2500 K and 5000 K are included as black dots.

Finally, aiming to obtain multicolor UC emission, we synthesize YF_3_:Yb^3+^,Ho^3+^ and YF_3_:Yb^3+^,Tm^3+^ nanoparticles, as described in the Methods and Materials section. Ho^3+^- and Tm^3+^-doped nanoparticles feature similar size and morphology than Er^3+^-doped ones. Full details are provided in the Methods and Materials section, as well as in Figure S6 in the Supporting Information. We follow the procedure discussed in the Methods and Materials section to prepare YF_3_:Yb^3+^,Ho^3+^ and YF_3_:Yb^3+^,Tm^3+^ pastes, deposit nanophosphor films, anneal them to achieve the proper crystalline phase, and infiltrate them with PMMA. Notice that we choose the experimental conditions that yield most efficient UCPL for Er^3+^-doped nanophosphors to process Ho^3+^- and Tm^3+^-doped materials, because structural properties are expected to be rather independent of the particular choice of active cation. As a result, both rigid and flexible versions of oxyfluoride-based nanophosphor thin films doped with (Yb^3+^, Er^3+^), (Yb^3+^, Ho^3+^), and (Yb^3+^, Tm^3+^) are achieved. Figure 5a shows the UCPL spectra of the different ∼5-μm-thick nanophosphor films infiltrated with PMMA. Time-dependent UCPL measurements are included in Figure S7 in the Supporting Information. Bright red emission is observed for (Yb^3+^, Er^3+^)-doped composite films, as illustrated in Figure 5b. (Yb^3+^, Ho^3+^)-doped sample feature bands of UCPL in the red and green parts of the electromagnetic spectrum associated, respectively, to the transition from ^5^F_4_ and ^5^S_2_ levels to the ground state ^5^I_8_, and the transition from ^5^F_5_ to the ground state of Ho^3+^ - see Figure 5a.^66,67^ The combination of green and red emission yields orange UC, as depicted in Figure 5c. (Yb^3+^, Tm^3+^)-doped layers present UCPL bands in the NIR and the blue corresponding, respectively, to the transitions from ^1^G_4_ to ^3^F_4_ and from ^3^F_3_ to ^3^H_6_ states, and to the transitions from ^1^D_2_ to ^3^F_4_ and from ^1^G_4_ to ^3^H_6_.^50,65^ Since the human eye is not sensitive to NIR light, UCPL of Tm^3+^ is perceived as blue, as it can be observed in Figure 5d. The comparison between the UCPL intensity of the different films prepared highlights significant differences in UC efficiency. Specifically, Er^3+^ yields the brightest UCPL partly because the ^2^F_5/2_ energy level of Yb^3+^ matches better with the energy level of the metastable intermediate excited state of Er^3+^ than that of Ho^3+^ or Tm^3+^.^2,50,74^

The versatility of our method allows the demonstration of white light upconversion by combining the emission of individual layers in a stack. To prove our point, a PMMA-infiltrated (Yb^3+^, Ho^3+^)-doped nanophosphor film is coated with a (Yb^3+^, Tm^3+^)-based sticker to yield the UCPL spectrum shown in Figure 5e. Similarly, it is also possible to repeat the process and coat the Ho^3+^-doped film with a second Tm^3+^-based layer to increase the contribution of blue light to the mixture. UCPL spectra attained from the ∼10 μm- and ∼15 μm-thick stacks are plotted in Figure 5e. The mixture of the blue emission of Tm^3+^-doped film and the orange emission of Ho^3+^ ions yields white light—see Figure 5f—with chromaticity coordinates that lie in the achromatic region of the chromaticity diagram shown in Figure 5g. In particular, warm white light with correlated color temperatures between 2500 K and 4000 K can be achieved, depending on the combination of upconverting stickers. This approach presents a clear advantage over the standard route to achieve multicolored UC emission, which consists of doping a given host with more than one active ion. It prevents cross-relaxation, deleterious for UCPL, and provides an easy way to tune the chromaticity of the UC emission.

## Conclusions

We have developed a simple preparation method to achieve highly transparent, self-standing flexible upconverting nanophosphor films based on an oxyfluoride matrix doped with rare-earth (RE) cations. Colloidally stable and uniform RE-doped YF_3_ nanoparticles were synthesized and used to prepare dispersions with which to deposit films of controlled thickness and high optical quality (i.e., scattering free). Thermal annealing allows improving the crystallinity of the host while inducing a phase transformation from YF_3_ to oxyfluoride. In particular, the analysis of the upconversion photoluminescence reveals that the highest upconversion efficiency is obtained for films annealed at 450 °C, which features an orthorhombic yttrium oxyfluoride crystal phase. Also, we demonstrate a clear correlation between photophysical and structural properties, with distinct decay rates associated with cations embedded in different crystal lattices. In addition, the infiltration of the nanopshosphor pore network with a polymer allows the fabrication of adaptable upconverting oxyfluoride coatings. Finally, we have demonstrated that it is possible to combine upconverting stickers that emit blue and orange to yield tunable warm white light. Our results pave the way to the development of highly versatile coatings, based on efficient upconverting nanoparticles.

## Methods and Materials

### Chemicals

Yttrium(III) chloride hexahydrate (YCl_3_**·**6H_2_O, Sigma–Aldrich, 99.9%), erbium(III) chloride hexahydrate (ErCl_3_**·**6H_2_O, Sigma–Aldrich, 99.9%), holmium(III) chloride hexahydrate (HoCl_3_**·**6H_2_O, Sigma–Aldrich, 99.9%), thulium(III) chloride hexahydrate (TmCl_3_**·**6H_2_O, Sigma–Aldrich, 99.9%), ytterbium(III) chloride hexahydrate (YbCl_3_**·**6H_2_O, Aldrich, 99.9%) were selected as lanthanide (Ln) precursors. 1-Butyl, 3-methylimidazolium tetrafluoroborate, ([BMIM]BF_4_, C_8_H_15_BF_4_N_2_, Fluka, > 97%), was used as fluoride source and diethylene glycol (DEG) (Sigma–Aldrich, 99%) as solvent. Ethyl cellulose (Sigma–Aldrich, powder) was used as organic binder and α-terpineol (SAFC, ≥96%) as a solvent in the paste preparation. Poly(methyl methacrylate) (PMMA, Alfa Aesar, powder) was chosen as a support material to prepare a flexible version of the nanophosphor coating.

### Nanoparticle Synthesis

Yttrium fluoride nanoparticles containing 20% ytterbium and 2% of erbium were synthesized following a procedure reported elsewhere.^44^ Briefly, 1.872 mmol of YCl_3_ and 0.48 mmol of YbCl_3_ were dissolved together in 105.6 mL of DEG under magnetic stirring and heating at 70 °C, while 0.048 mmol of ErCl_3_ was dissolved in 12 mL of DEG in another vial at the same condition. After dissolving, ErCl_3_ was added to a solution containing YCl_3_ and YbCl_3_ precursors, then the solution was cooled to room temperature. Consequently, 2.4 mL of [BMIM]BF_4_ was admixed keeping the magnetic stirring for few minutes at room temperature to obtain a homogeneous mixture. The final solution was introduced in an oven at 120 °C and heated at this temperature for 15 h. After aging, the resulting dispersion was cooled to room temperature. The nanoparticles were centrifuged and cleaned three times with absolute ethanol, then dispersed in methanol for the preparation of colloidal suspensions and pastes. The yttrium fluoride nanoparticles doped with of 20% ytterbium and 0.5% of holmium or 0.5% of thulium were synthesized adjusting the amounts of HoCl_3_ or TmCl_3_ precursors and following the same procedure.

### Nanophosphor Paste

The preparation of a paste from nanophosphor particles was performed following a procedure described elsewhere.^45^ In brief, nanophosphor particles, with mass *m*_np_, were dispersed in 120 mL of methanol and sonicated with a tip sonicator for 10 min to minimize the aggregation of particles. An amount of 0.3·*m*_np_ of ethyl cellulose was added to nanophosphor particle suspension, followed by a process of magnetic stirring for 5 min and tip sonication for another 5 min. Subsequently, an amount of 4·*m*_np_ of α-terpineol was added, following the same sequence of magnetic stirring and tip sonication. Finally, a viscous paste was obtained by evaporating methanol at reduced pressure.

### Nanophosphor Films

*Thin nanophosphor films* (thickness below ∼1 μm) were obtained by spin-coating of the nanoparticle suspensions in methanol.^47−49^ Film thickness can be easily tuned by changing the suspension concentration or the spin coating parameters, being generally thicker layers achieved using more concentrated suspensions or lower speeds. Thicker film can also be attained repeating the spin coating process. Further details are provided in the Supporting Information. *Thick nanophosphor films* (thickness between ∼1 μm and ∼15 μm) were fabricated using a blade coating method. A fraction of nanophosphor paste was placed on a substrate (glass, quartz, or glass/SiO_2_-dense) and extended over it (see Figure 1b).^45^ The film thickness was controlled by the number of spacers. Resulting films were annealed for 6 or 10 h in a hot plate at temperatures ranging from 400 °C to 550 °C with a rate of 2 °C per minute.

### Flexible Nanophosphor Coating

Poly(methyl methacrylate) (PMMA) was selected as support material for the flexible nanophosphor coating. PMMA solutions with concentrations of 5 and 8 wt % were obtained by dissolving PMMA in anisole. First, a sacrificial SiO_2_-dense layer with a thickness of ∼150 nm was deposited over the substrate via a two-step spin coating process at 500 rpm for 20 s and at 2000 rpm for 60 s, followed by the annealing at 500 °C in a hot plate for 30 min. Then, we deposited the nanophosphor film following the method described above. This step also includes thermal processing if needed. For the polymer infiltration, 5 wt % PMMA solution was first infiltrated onto annealed nanophosphor films. A 8 wt % PMMA solution was next deposited on the resulted films with the same spin coating parameters. After infiltrating PMMA, samples were dried at 60 °C for at least 1 h. Dried samples were immersed in a 1% hydrofluoric acid (HF, Fluka, 48%) solution in Milli-Q water (Millipore, Bedford, MA) for etching the sacrificial SiO_2_ dense layer. After 60 min, a flexible coating was detached from substrate and washed abundantly in water to remove residual HF.

### Morphological and Structural Characterization

The shape and size of synthesized nanophosphor particles were revealed by means of transmission electron microscopy (TEM) (Philips, Model 200CM). Top view and cross section of annealed nanophosphor particle films on rigid substrates were obtained using Scanning Electron Microscopy (SEM) (Hitachi Model S4800 microscope). XRD patterns were attained using Panalytical X’pert Pro. In particular, as-deposited (YF_3_:Yb^3+^Er^3+^) nanophosphor films were measured in a grazing angle configuration, whereas annealed films were measured in standard configuration.

### Optical Characterization

Total transmittance (*T*_tot_) and total reflectance (*R*_tot_) was collected using an UV-vis-IR spectrophotometer Cary 7000 equipped with an integrating sphere. The absorptance was calculated using the following equation:
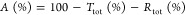
Photoluminescence (PL) and PL decay measurements were performed using a spectrofluorometer (Edinburgh Instruments, Model FLS1000). As an excitation source, we used a 980 nm laser (2 W of optical power) operating at maximum power in continuous mode for static UC measurements and in pulsed mode (repetition rate of 250 Hz and pulse width of 360 μs) for time-dependent PL intensity analysis. A computer control neutral density filter wheel was applied before the sample to adjust the power of the laser, to study the power dependence of PL intensity.

### Lifetime Analysis

The lifetime results were processed using FAST software from Edinburgh, taking into account the instrumental response function using the *Exponential Component Analysis (Reconvolution)* model.

## Supporting Information Available

Details on the structural, optical, and photophysical properties of upconverting nanophosphors and coatings (PDF)
