# Supplementary material for: Highly Versatile Upconverting Oxyfluoride-Based Nanophosphor Films
Source: ACS Appl Mater Interfaces. 2021 Jun 18;13(25):30051–60. doi: 10.1021/acsami.1c07012 (PMC8251696; doi:10.1021/acsami.1c07012)
Supplement: Supplementary file 1 — am1c07012_si_001.pdf [file am1c07012_si_001.pdf]

Supporting information of

Highly versatile up-converting oxyfluoride-based nanophosphor films

T. Tuyen Ngo,† Elena Cabello-Olmo,† Encarnación Arroyo, Ana I. Becerro,* Manuel Ocaña, Gabriel Lozano,* Hernán Míguez

Instituto de Ciencia de Materiales de Sevilla, Consejo Superior de Investigaciones Científicas-Universidad de Sevilla, Américo Vespucio 49, 41092, Sevilla, Spain

† Authors with equal contribution.

* anieto@icmse.csice.es; g.lozano@csic.es

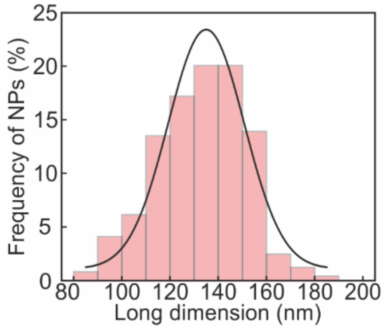

Figure S1. Long dimension distribution histogram of YF_3:$Yb^{3+}Er^{3+}$ nanoparticles. The mean size is 134 nm (σ = 19 nm).

Thin films were deposited from suspensions of (Yb^{3+}, Er^{3+})-doped nanophosphors in methanol (8.2% by wt.). First, a thin film (~30 nm) of TiO_2 was deposited on the quartz substrate by dip coating (immersion speed 400 mm/minute, withdrawal speed 120 mm/minute, up waiting 30 seconds, down waiting 10 seconds) in order to improve the uniformity of the nanophosphor films deposited atop. Then, the dispersion of nanophosphors was deposited by spin coating using 180 µL with an acceleration ramp of 11 340 rpm s^{-1} for 1 minute and a final rotation speed of 1500 rpm. Please, notice that acceleration is very high to ensure that the final rotation speed is reached within a fraction of a second. These conditions have been shown to be effective in producing uniform films. This procedure was repeated 6 times to obtain thicker films. As a result, transparent films with a thickness around 1.3 µm were obtained. Finally, the samples were annealed with a rate 1 ºC per minute and a plateau of 2 hours at 400 ºC.

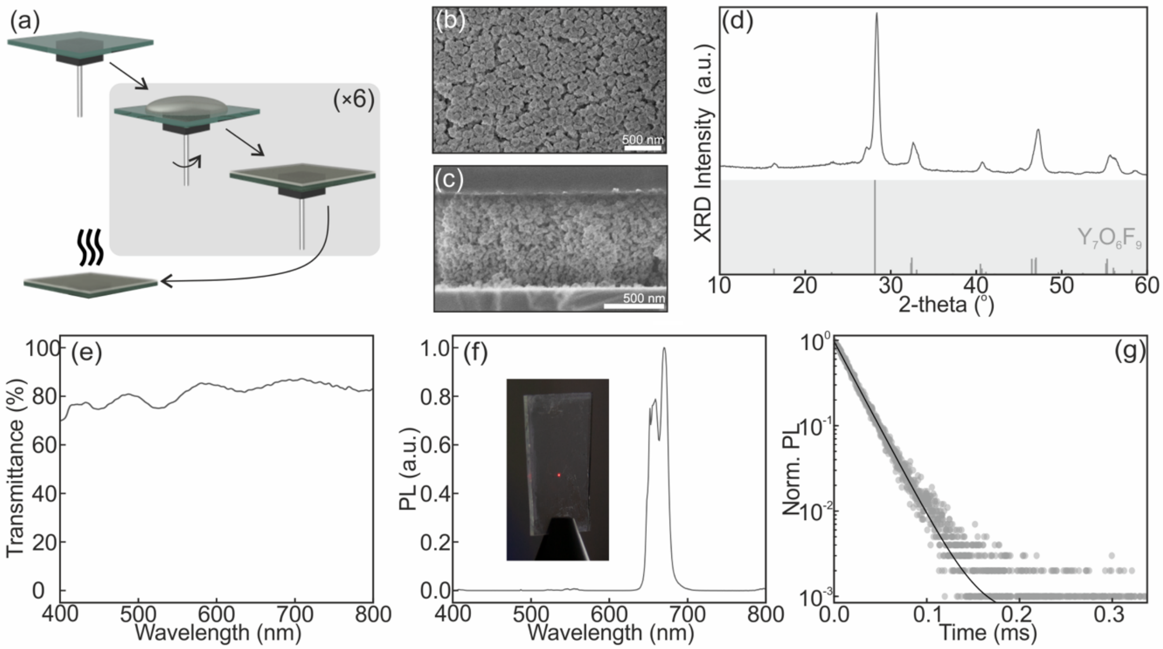

Figure S2. (a) Schematic processing of nanophosphor thin film preparation by spin coating. (b-c) Scanning electron micrographs of a top view (b) and a cross section (c), of a representative film after annealing at 400 ºC. (d) Diffractogram of the sample along with the reference pattern for the $Y_7O_6F_9$ phase (PDF 01-070-0867). (e) Experimental ballistic transmittance spectrum. (f) Experimental photoluminescence spectrum of the film under 980 nm excitation. Picture of the up-conversion emission of the film is shown as inset. (g) Time-dependent PL of the most intense transition of Er^{3+} ($^4F_{9/2} - {}^4I_{15/2}$) and the fitting to a one-exponential decay (τ =21.4 µs).

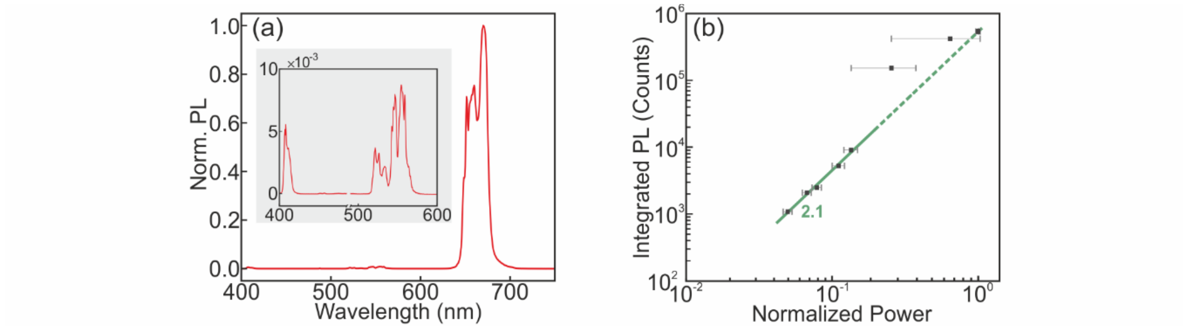

Figure S3. (a) Up-conversion photoluminescence (UCPL) spectrum of a (Yb^{3+}, Er^{3+}) nanophosphor thick film annealed at 450 ºC and for 6 hours. The 400-600 nm range is shown in the inset. (b) Integrated UCPL intensity of the green band (i.e. 500 – 600 nm range) as a function of the excitation power.

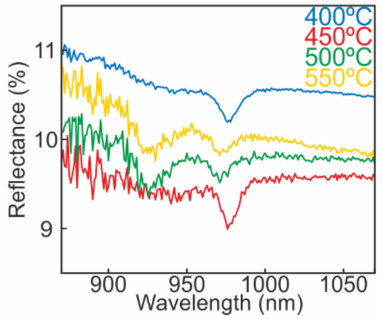

Figure S4. Total reflectance spectra of (Yb^{3+}, Er^{3+}) nanophosphor thick films annealed at temperatures ranging from 400 ºC to 550 ºC for 6 hours.

Figures S5a-b show the XRD patterns of (Yb^{3+}, Er^{3+}) nanophosphor films annealed at different conditions. The reflections of the nanophosphor thick films annealed at 450 ºC and 500 ºC for 6 hours are in agreement with pure orthorhombic $Y_7O_6F_9$ and pure rhombohedral YOF, respectively. The film annealed at 450 ºC for 10 hours shows, however, two different sets of reflections indicating the coexistence of both orthorhombic $Y_7O_6F_9$ and rhombohedral YOF phases. Figure S5c shows the effect of longer annealing time on the Up-conversion photoluminescence intensity.

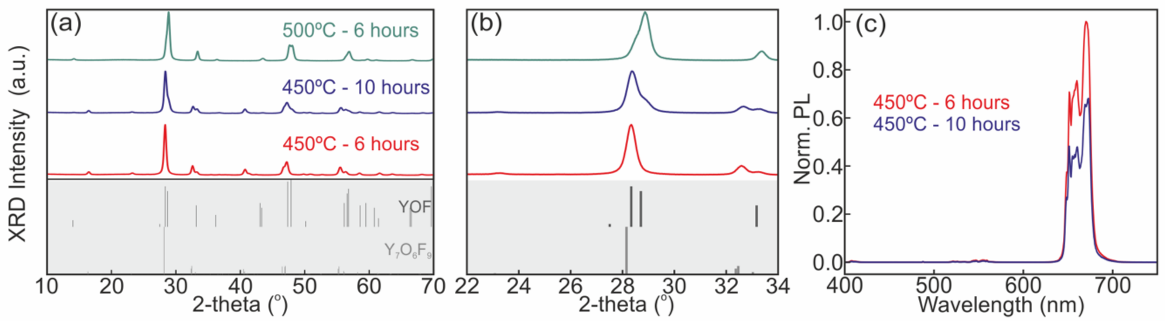

Figure S5. (a) X-ray diffraction patterns of (Yb^{3+}, Er^{3+}) nanophosphor thick films annealed at different conditions. (b) Detail of the patterns in the angular range comprised between 22º and 34º. $Y_7O_6F_9$ (PDF 01-070-0867) and YOF (PDF 00-025-1012) reference patterns are included. (c) Up-conversion photoluminescence spectra of (Yb^{3+}, Er^{3+}) nanophosphor thick films annealed at 450 ºC for 6 hours and 10 hours.

YF_3:Yb^{3+}, Ho^{3+} and YF_3:Yb^{3+}, Tm^{3+} nanoparticles were synthesized following a similar synthetic route used for YF_3:Yb^{3+}, Er^{3+} nanoparticles with lower content of Ho^{3+} or Tm^{3+}. Particles show very similar morphology to that of YF_3:Yb^{3+}, Er^{3+} nanoparticles (see Figure 1a) with slightly longer mean sizes, 142 nm for YF_3:Yb^{3+}, Ho^{3+} nanoparticles and 139 nm for YF_3:Yb^{3+}, Tm^{3+} nanoparticles.

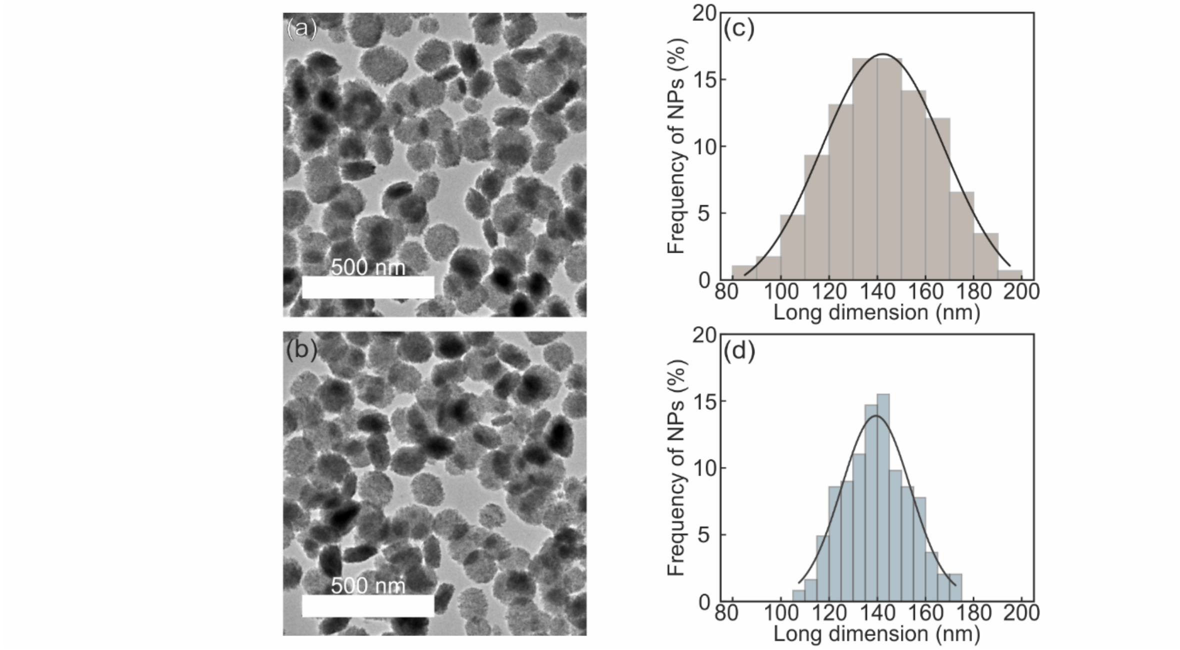

Figure S6. TEM images (a-b) and size distribution histogram (c-d) of (a-c) $YF_3:Yb^{3+}Ho^{3+}$ and (b-d) $YF_3:Yb^{3+}Tm^{3+}$ nanophosphor particles. The mean long dimensions are 142 nm (σ = 25nm) and 139 nm (σ = 19 nm), respectively.

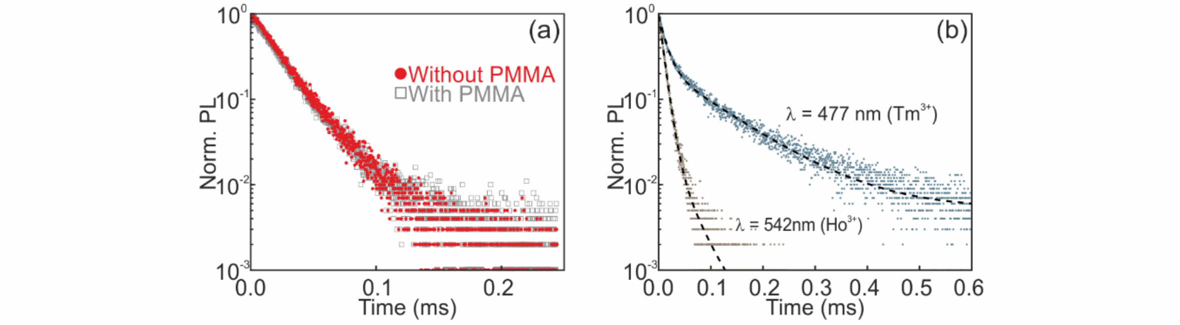

Figure S7. (a) Time-dependent PL of (Yb^{3+}, Er^{3+}) thick films before (dots) and after (squares) infiltrating PMMA. (b) Time-dependent PL of (Yb^{3+}, Ho^{3+}) and (Yb^{3+}, Tm^{3+}) thick films (distinguished as Ho^{3+} and Tm^{3+}) infiltrated with PMMA and measured at 542 and 477 nm, respectively. Experimental data and fitted curves are plotted with dots and dashed lines, respectively. All samples were excited with 980-nm pulse laser.

Samples	τ_1 (μs)	w_1 (%)	τ_2 (μs)	w_2 (%)	τ_{aver} (μs)
(Yb, Ho) / PMMA	8.5	98.6	32.7	1.4	9.8
(Yb, Tm) / PMMA	13.8	92.6	100	7.4	45.3

Table S1. Fitted parameters obtained from the PL decay curves plotted in the Figure S7b
